# Association of the advanced lung cancer inflammation index (ALI) with immune checkpoint inhibitor efficacy in patients with advanced non-small-cell lung cancer

**DOI:** 10.1016/j.esmoop.2021.100254

**Published:** 2021-09-01

**Authors:** G. Mountzios, E. Samantas, K. Senghas, E. Zervas, J. Krisam, K. Samitas, F. Bozorgmehr, J. Kuon, S. Agelaki, S. Baka, I. Athanasiadis, L. Gaissmaier, M. Elshiaty, L. Daniello, A. Christopoulou, G. Pentheroudakis, E. Lianos, H. Linardou, K. Kriegsmann, P. Kosmidis, R. El Shafie, M. Kriegsmann, A. Psyrri, C. Andreadis, E. Fountzilas, C.-P. Heussel, F.J. Herth, H. Winter, C. Emmanouilides, G. Oikonomopoulos, M. Meister, T. Muley, H. Bischoff, Z. Saridaki, E. Razis, E.-I. Perdikouri, A. Stenzinger, I. Boukovinas, M. Reck, K. Syrigos, M. Thomas, P. Christopoulos

**Affiliations:** 1 Fourth Oncology Department and Clinical Trials Unit, Henry Dunant Hospital Center, Athens, Greece; 2 Second Oncology Department, Metropolitan Hospital, Pireaus, Athens, Greece; 3 Thoraxklinik and National Center for Tumor Diseases at Heidelberg University Hospital, Heidelberg, Germany; 4 7th Pneumonology Department ‘Sotiria’ Hospital, Athens, Greece; 5 Institute of Medical Biometry and Statistics, Heidelberg University Hospital, Heidelberg, Germany; 6 Department of Medical Oncology, University of Irakleion School of Medicine, Iraklion, Greece; 7 Department of Medical Oncology, Interbalkan Medical Center, Thessaloniki, Greece; 8 Department of Medical Oncology, ‘Mitera’ Hospital, Athens, Greece; 9 Translational Lung Research Center Heidelberg, German Center for Lung Research (DZL), Heidelberg, Germany; 10 Department of Medical Oncology, General Hospital of Patras ‘Agios Andreas’, Patras, Greece; 11 Department of Medical Oncology, University of Ioannina School of Medicine, Ioannina, Greece; 12 Department of Medical Oncology, ‘Metaxa’ Cancer Hospital, Pireaus, Greece; 13 Fourth Oncology Department, Metropolitan Hospital, Pireaus, Athens, Greece; 14 Department of Hematology, Oncology and Rheumatology, University Hospital Heidelberg, Heidelberg, Germany; 15 Second Oncology Department, ‘Hygeia’ Hospital, Athens, Greece; 16 Department of Radiation Oncology, Heidelberg University Hospital, Heidelberg, Germany; 17 Department of Medical Oncology, ‘Attikon’ University Hospital, Athens, Greece; 18 Third Department of Medical Oncology, ‘Theageneion’ Cancer Hospital, Thessaloniki, Greece; 19 Department of Medical Oncology, ‘Euromedica’ Clinic, Thessaloniki, Greece; 20 Department of Medical Oncology, ‘Asclepius’ Clinic, Iraklion, Greece; 21 Third Department of Medical Oncology, Hygeia Hospital, Athens, Greece; 22 Department of Medical Oncology, ‘Achilopouleio’ General Hospital of Volos, Volos, Greece; 23 Department of Medical Oncology, ‘Bioclinica’ Hospital, Thessaloniki, Greece; 24 LungenClinic Großhansdorf GmbH, Großhansdorf, Germany; 25 Airway Research Center North, German Center for Lung Research, Großhansdorf, Germany; 26 Department of Medical Oncology, Sotiria General Hospital of Athens, Athens, Greece

**Keywords:** advanced lung cancer inflammation index, immunotherapy, non-small-cell lung cancer, PD-L1, neutrophil-to-lymphocyte ratio

## Abstract

**Background:**

The advanced lung cancer inflammation index [ALI: body mass index × serum albumin/neutrophil-to-lymphocyte ratio (NLR)] reflects systemic host inflammation, and is easily reproducible. We hypothesized that ALI could assist guidance of non-small-cell lung cancer (NSCLC) treatment with immune checkpoint inhibitors (ICIs).

**Patients and methods:**

This retrospective study included 672 stage IV NSCLC patients treated with programmed death-ligand 1 (PD-L1) inhibitors alone or in combination with chemotherapy in 25 centers in Greece and Germany, and a control cohort of 444 stage IV NSCLC patients treated with platinum-based chemotherapy without subsequent targeted or immunotherapy drugs. The association of clinical outcomes with biomarkers was analyzed with Cox regression models, including cross-validation by calculation of the Harrell's C-index.

**Results:**

High ALI values (>18) were significantly associated with longer overall survival (OS) for patients receiving ICI monotherapy [hazard ratio (HR) = 0.402, *P* < 0.0001, *n* = 460], but not chemo-immunotherapy (HR = 0.624, *P* = 0.111, *n* = 212). Similar positive correlations for ALI were observed for objective response rate (36% versus 24%, *P* = 0.008) and time-on-treatment (HR = 0.52, *P* < 0.001), in case of ICI monotherapy only. In the control cohort of chemotherapy, the association between ALI and OS was weaker (HR = 0.694, *P* = 0.0002), and showed a significant interaction with the type of treatment (ICI monotherapy versus chemotherapy, *P* < 0.0001) upon combined analysis of the two cohorts. In multivariate analysis, ALI had a stronger predictive effect than NLR, PD-L1 tumor proportion score, lung immune prognostic index, and EPSILoN scores. Among patients with PD-L1 tumor proportion score ≥50% receiving first-line ICI monotherapy, a high ALI score >18 identified a subset with longer OS and time-on-treatment (median 35 and 16 months, respectively), similar to these under chemo-immunotherapy.

**Conclusions:**

The ALI score is a powerful prognostic and predictive biomarker for patients with advanced NSCLC treated with PD-L1 inhibitors alone, but not in combination with chemotherapy. Its association with outcomes appears to be stronger than that of other widely used parameters. For PD-L1-high patients, an ALI score >18 could assist the selection of cases that do not need addition of chemotherapy.

## Highlights

• ALI is prognostic and predictive for patients with advanced NSCLC treated with immunotherapy monotherapy, but not chemo-immunotherapy.
• Its association with outcomes is stronger than that of other parameters (PD-L1 TPS, NLR, lung immune prognostic index, EPSILoN).
• For PD-L1-high patients, an ALI score >18 could assist the selection of cases that do not need addition of chemotherapy.

## Introduction

The advent of immunotherapy with checkpoint inhibitors (ICI) has heralded a new era in the therapeutic landscape of advanced non-small-cell lung cancer (NSCLC).^1^ The anti-programmed cell death protein 1 (PD-1) monoclonal antibody pembrolizumab has been shown to improve survival as monotherapy in the first-line setting of patients with advanced NSCLC and expression of programmed death-ligand 1 (PD-L1) in >50% of cancer cells^2^ and in combination with platinum-based chemotherapy in all patients with advanced disease irrespective of PD-L1 immunohistochemical expression.^3^^,^^4^ The anti-PD-L1 monoclonal antibody atezolizumab also improves survival in the first-line setting, either as monotherapy for patients with PD-L1 expression on immune or tumor cells ≥50%, or in combination with platinum-based chemotherapy^5^^,^^6^ with or without the anti-angiogenic agent bevacizumab.^7^ In addition, several ICIs have been associated with improved survival as second-line treatment, compared with chemotherapy with docetaxel, in patients previously treated with platinum-based chemotherapy.^8^, ^9^, ^10^, ^11^ Despite these impressive advances, a considerable number of patients still do not respond to immunotherapy, including many cases with PD-L1-positive tumors. Since PD-L1 is a suboptimal predictor of ICI efficacy, several other potential biomarkers have been proposed, including tumor mutational burden,^12^ infiltration of the tumor stroma by T-lymphocytes and other immune cell effectors^13^ and molecular signatures involving clusters of genes related to inflammation, such as interferon-γ cluster gene expression.^14^ All the above methods have yielded inconclusive results to date, are technically demanding, and costly.^15^

Systemic inflammation of the host has been proposed as a hallmark of cancer, associated with activation of oncogenic signaling pathways, leading to cancer dissemination, growth, and metastasis.^16^ It is well established that systemic inflammation is a poor prognostic factor, typically associated with malnutrition, hypoalbuminemia, weight loss, and other features of cancer cachexia.^17^ A number of related parameters have been evaluated as potential biomarkers of systemic inflammation in patients with cancer, including C-reactive protein (CRP), lactate dehydrogenase (LDH), circulating white blood cells (WBC), absolute neutrophil count (ANC), neutrophil-to-lymphocyte ratio (NLR) and derived NLR [dNLR; ANC/(WBC concentration−ANC)].^17^ A number of clinical algorithms have also been developed, based on various combinations of the aforementioned variables, such as the lung immune prognostic index (LIPI, comprising dNLR and LDH) and the EPSILoN index [Eastern Cooperative Oncology Group (ECOG) performance status (PS), smoking, liver metastases, LDH, NLR].^18^ In 2013, the concept of advanced lung cancer inflammation index (ALI) was introduced and was first determined to be an effective prognostic index in metastatic NSCLC.^19^ ALI is calculated from the following equation:

$$\text{ALI}=\frac{\text{Body}\;\text{mass}\;\text{index}\left({\text{kg/m}}^{2}\right)\times \text{serum}\;\text{albumin}\left(\text{g/dl}\right)}{\text{NLR}}.$$

Comprising indicators of nutritional and inflammatory status of the host, ALI has the potential to reflect the systemic inflammation and cachexia provoked by cancer, rendering it an attractive candidate biomarker of immunotherapy efficacy in NSCLC patients.

Although a low ALI score (<18) has been shown to be an independent poor prognostic factor in patients with advanced NSCLC,^19^^,^^20^ its predictive value in patients receiving ICIs is unknown. We hypothesized that low ALI values could be associated with resistance to ICIs and represent a simple tool to predict immunotherapy efficacy in patients with advanced NSCLC. We also set out to assess the predictive capacity of ALI compared with other potential biomarkers of ICI efficacy, including PD-L1, NLR, LIPI, and EPSILoN.

## Patients and methods

### Study population and data collection

We conducted a multicenter, retrospective, cross-sectional study of NSCLC patients treated with either PD-L1 inhibitors alone in any treatment line (cohort ‘A’), or with first-line chemo-immunotherapy (cohort ‘B’) at 25 institutions in Greece and Germany. Eligible were all cases at each participating center, for which data about the ALI and other parameters with potential influence on ICI efficacy were available. All patients were diagnosed according to the current World Health Organization (WHO)/International Association for the Study of Lung Cancer (IASLC) guidelines^21^ and had been tested negative for *EGFR* and *ALK* alterations. An additional, control cohort ‘C’, including all NSCLC patients who had received first-line platinum-based chemotherapy without subsequent targeted or ICI drugs and had available pertinent data, was assembled at the Heidelberg University Hospital in order to discern predictive from prognostic effects.

Basic clinicopathological and somatometric data, complete blood counts, biochemical variables and serum albumin levels at baseline (within 21 days from the first treatment) were extracted from medical records and used to calculate the ALI score and NLR as published.^19^^,^^22^ The ALI and NLR were dichotomized at the bibliographic cut-offs of 18 and 5, respectively, which corresponded approximately to the median values of our untreated cohort AB patients (median ALI 19.5 and median NLR 4.8). Other indexes (LIPI, EPSILoN) were calculated according to the respective publications.^18^^,^^23^ Immunohistochemical PD-L1 expression was analyzed locally, using monoclonal antibodies and platforms validated at each center, reported as ‘tumor proportion score’ (TPS), and divided in three categories: negative (<1%), low (1%-49%), and high (≥50%). Overall survival (OS) was calculated from the start of treatment with PD-L1 inhibitors (experimental cohort) or chemotherapy (control cohort) to the date of death for any reason or last date of follow-up, if patients were alive. The ‘time-on-treatment’ (TOT) was defined as the time from start of PD-L1 inhibitor (experimental cohort) or chemotherapy (control cohort) until treatment discontinuation or death, and was used as a proxy for the duration of benefit from therapy. Tumor responses were verified by review of radiological images, i.e. chest/abdomen computed tomography (CT) and brain magnetic resonance imaging (MRI) every 6-12 weeks, by the investigators based on the RECIST v.1.1 principles. Objective response rate (ORR) refers to the sum of complete response (CR) + partial response (PR), and disease control rate (DCR) to the sum of CR + PR + stable disease. For progression-free survival (PFS), the progression date was calculated from the date of treatment initiation and verified by the investigators through a review of radiologic images, i.e. chest/abdomen CT and brain MRI every 6-12 weeks, without formal RECIST re-evaluation, as several studies have demonstrated very good agreement between real-world and RECIST-based PFS assessments.^24^^,^^25^

The study was approved by the Institutional Review Board of the Henry Dunant Hospital in Athens, Greece and by the Ethics Committee of the Heidelberg University Hospital (S-145/2017). Since this was a non-interventional, retrospective study, informed consent was obtained whenever possible, but was not required for every participant. Results of the study are reported according to the STROBE guidelines for observational studies (available at https://www.equator-network.org/reporting-guidelines/strobe).

### Statistical analysis

Comparisons between patient characteristics and clinicopathological data were carried out using the chi-square test for categorical and the Kruskal–Wallis test for numerical variables. The 95% confidence intervals (95% CI) for proportions were calculated using the modified Wald method.^26^ Survival analyses were carried out according to the Kaplan–Meier method with the log-rank test or Cox proportional hazards models, including calculation of Harrell's C-index in both cohorts for validation of results *via* 10-fold cross-validation. Variables included in the final multivariate model were selected based on the results of univariate analyses and their clinical relevance to the outcome of interest, which in our case were age (in years), sex, first versus subsequent-line treatment, ECOG PS (>1/1/0), and PD-L1 TPS (0/1-49/50+). Statistical analysis was carried out at the Institute of Biometry of the Heidelberg University Hospital using R version 4.0 together with the packages rms and CsChange. *P* values below 0.05 (two-sided) were considered statistically significant. With the sample size of *n* = 460 patients receiving ICI monotherapy at hand (of which 201 are in the subgroup with low ALI, and 259 are in the subgroup with high ALI), a two-sided test of whether the hazard ratio (HR) for ALI is equal to one achieves 80% power at a 5% significance level when HR = 0.68 (assuming event probabilities of 0.63 for low ALI and 0.34 for high ALI, calculation done using PASS version 16.0.3).

## Results

### Patient characteristics

**Figure 1:**
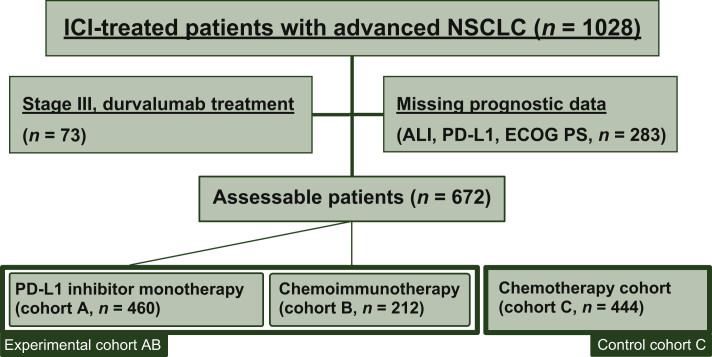
Flow chart of the study cohorts. ALI, advanced lung cancer inflammation index; ECOG PS, Eastern Cooperative Oncology Group performance status; ICI, immune checkpoint inhibitor; NSCLC, non-small-cell lung cancer; PD-L1, programmed death-ligand 1.

Overall, 672 patients treated with immunotherapy alone or in combination with chemotherapy (experimental cohort ‘AB’) and 444 patients treated with chemotherapy (control cohort ‘C’) could be recruited, with their characteristics summarized in Table 1. Median age was 65 and 63 years, respectively, with a predominance of male patients (69% and 67%). The experimental cohort AB consisted of two subcohorts: cohort A (*n* = 460) including patients who received PD-L1 monotherapy in various treatment lines, and cohort B (*n* = 212) with patients who received first-line chemo-immunotherapy (Figure 1 and Table 1). In the entire cohort AB, most patients had an ECOG PS of 0/1 (87%), PD-L1-positive tumors (81% with TPS at least 1%), and adenocarcinomas (73%), In the same cohort (AB), the ORR was 35%, the DCR 61%, the median TOT 5.8 months, and the median OS 18.6 months (Table 1). Chemo-immunotherapy was administered in the first line only and was associated with a younger patient age, better ECOG PS, higher ORR and DCR, as well as longer TOT, PFS, and OS (Table 1). Besides, in the PD-L1 monotherapy subcohort there was a higher percentage of PD-L1-high tumors and squamous cell carcinomas (Table 1).

**Table 1:** Characteristics of study patients

	Experimental cohort (AB, *n* = 672)	PD-L1 inhibitor monotherapy (A, *n* = 460)	Chemo-immunotherapy (B, *n* = 212)	Control cohort (C, *n* = 444)	*P* value across A/B/C
Age at diagnosis, years, median (SD)	65 (13)	67 (10)	64 (10)	63 (9)	0.001
Sex, % male (*n*)	69 (461)	70 (324)	65 (137)	67 (298)	0.283
ECOG PS, % (*n*)					
0	37 (246)	34 (159)	41 (87)	51 (224)	0.001
1	50 (333)	51 (233)	47 (100)	46 (205)	0.381
≥2	14 (93)	15 (68)	12 (25)	3 (15)	0.001
Histology, % (*n*)					
Adenocarcinoma	74 (494)	69 (317)	84 (177)	72 (322)	0.001
Squamous carcinoma	22 (147)	26 (118)	14 (29)	19 (84)	0.001
Other	4 (31)	5 (25)	3 (6)	9 (38)	0.012
PD-L1 expression, % (*n*)					
Negative (TPS <1%)	19 (129)	18 (82)	22 (47)	—	0.184
TPS 1-49%	42 (279)	37 (169)	52 (110)	—	0.001
TPS ≥50%	39 (264)	45 (209)	26 (55)	—	0.001
Type of treatment, % (*n*)					
PD-L1 inhibitor alone	68 (460)			—	
Chemoimmunotherapy	32 (212)			—	
Chemotherapy	—			100 (444)	
Line of treatment, % (*n*)					
First line	55 (372)	35 (160)	100 (212)^a^	100 (444)	0.001
Second and beyond	45 (300)	65 (300)		—	
Baseline characteristics					
NLR, median (SD)	4.8 (8.1)	4.4 (8.9)	5.8 (6.3)	5.4 (6.3)	0.001
Height m, median (SD)	1.70 (0.09)	1.70 (0.09)	1.71 (0.10)	1.71 (0.09)	0.653
Weight kg, median (SD)	72 (16)	72 (16.5)	74 (16.1)	76 (16)	0.127
BMI, median (SD)	25 (4.8)	25 (5.0)	25 (4.5)	26 (5.1)	0.182
Albumin, median (SD)	3.9 (0.5)	3.8 (0.6)	4.1 (0.5)	3.9 (0.5)	0.001
ALI, median (SD)	19.5 (25)	20.6 (25)	17.6 (24)	18.3 (16.6)	0.051
Clinical outcome:					
ORR, % (95% CI)	35 (31-38)	31 (27-35)	43 (36-50)	na	0.003
DCR, % (95%CI)	61 (57-64)	56 (52-61)	75 (69-81)	na	0.001
TOT, median (95% CI), months	5.8 (4.6-7.0)	5.4 (4.3-6.5)	8.7 (5.0-12.4)	na	0.001
PFS, median (95% CI), months	5.3 (4.0-6.7)	3.3 (2.1-4.4)	8.0 (6.4-.9.7)	na	0.001
OS, median (95% CI), months	18.6 (14.5-22.6)	17.2 (13.3-21.1)	25.6 (9.3-41.9)	7.2 (6.5-7.9)	0.001

Statistical comparisons across cohorts (A, B, C) were performed with a chi-square for categorical, Kruskal-Wallis for numerical data, and logrank test for survival data.

ALI, advanced lung cancer inflammation index; BMI, body-mass index; CI, confidence interval; DCR, disease control rate; ECOG PS, Eastern Cooperative Oncology Group performance status; na, not available; NLR, blood neutrophil-to-lymphocyte ratio; ORR, overall response rate; OS, overall survival; PD-L1, programmed death-ligand 1; PFS, progression-free survival; TOT, time on treatment; TPS, tumor proportion score.

a Previous stage 3 disease in 31% (*n* = 67).

### ALI is a predictive and prognostic marker for PD-L1 inhibitor monotherapy stronger than NLR and PD-L1 TPS

**Figure 2:**
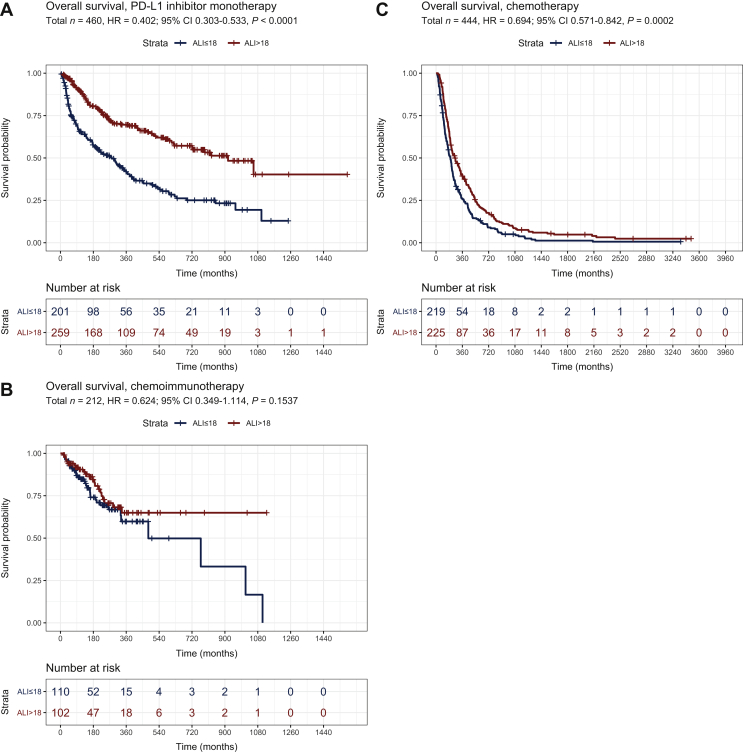

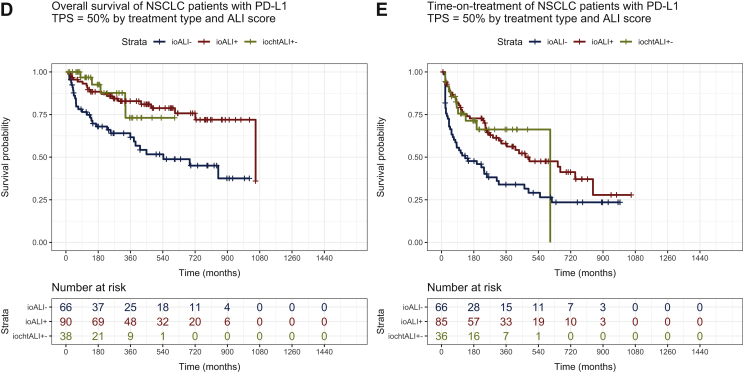
Survival of NSCLC patients according to ALI by type of treatment. (A) In patients receiving PD-L1 inhibitor monotherapy, median overall survival (OS) was 30.6 months [95% confidence interval (CI) 21.9-39.3 months] for patients with ALI >18 versus 9.2 months (95% CI 6.2-12.1 months) for patients with ALI ≤18 (logrank *P* = 3.07 × 10^−11^). (B) In patients receiving chemo-immunotherapy, median OS was not reached for patients with ALI >18 versus 16.0 months (95% CI 4.5-27.5 months) for patients with ALI ≤18 (logrank *P* = 0.1537). (C) In patients receiving chemotherapy, median OS was 8.6 months (95% CI 6.6-10.6 months) for patients with ALI >18 versus 6.6 months (95% CI 5.6-7.5 months) for patients with ALI ≤18 (logrank *P* = 0.0002). (D) In patients with PD-L1 TPS ≥50% receiving first-line PD-L1 inhibitor monotherapy, median OS was 35.2 months (95% CI 19.6-50.9) for patients with ALI >18 versus 18.1 months (CI 6.7-29.4 months) for patients with ALI ≤18 (logrank *P* = 0.0003). In patients receiving chemo-immunotherapy, median OS was not reached (logrank *P* = 0.24 with chemo-immunotherapy for ALI >18 versus ALI ≤18, Supplementary Figure S1A, available at https://doi.org/10.1016/j.esmoop.2021.100254). (Ε) In patients with PD-L1 TPS ≥50% receiving first-line PD-L1 inhibitor monotherapy, median time-on-treatment (TOT) was 15.6 months (95% CI 5.8-25.4 months) for patients with ALI >18 versus 4.4 (95% CI 0-9.2 months) for patients with ALI ≤18 (logrank *P* = 0.003). In patients receiving chemo-immunotherapy, median TOT was 20.2 months (logrank with chemo-immunotherapy for *P* = 0.94 for ALI >18 versus ALI ≤18, Supplementary Figure S1B, available at https://doi.org/10.1016/j.esmoop.2021.100254). ALI, advanced lung cancer inflammation index; HR, hazard ratio; NSCLC, non-small-cell lung cancer; PD-L1, programmed death-ligand 1; TPS, tumor proportion score.

High ALI values (>18) were significantly associated with longer OS for patients treated with PD-L1 inhibitor monotherapy (cohort A, HR = 0.40, 95% CI 0.30-0.53, *P* < 0.0001, *n* = 460, Figure 2A), but not for patients treated with chemo-immunotherapy (cohort B, HR = 0.62, CI 0.35-1.14, *P* = 0.1537, *n* = 212, Figure 2B). In the control cohort C of chemotherapy-treated patients, the association between ALI and OS was also significant, but less pronounced (HR = 0.69, CI 0.57-0.84, *P* = 0.0002, Figure 2C). In combined analysis of the cohorts A and C, there was a significant interaction between ALI and the type of treatment (for ICI versus chemotherapy, HR = 0.69 with *P* = 0.0009; for ALI >18 versus ALI ≤18, HR = 0.71 with *P* = 0.0006; for the interaction between ALI and the type of treatment, HR = 0.55 with *P* = 0.0004), suggesting that the ALI is both prognostic for NSCLC patients, and predictive for the benefit from PD-L1 inhibitor monotherapy. We further validated this finding by analyzing separately the cohort of patients from Germany as a training set and the cohort of patients from Greece as a validation set. The strong predictive effect of ALI for patients treated with PD-L1 inhibitor monotherapy was maintained in separate analyses of patients from Greece (*n* = 254) and Germany (*n* = 206), which served as training and validation cohorts, respectively, (OS HR = 0.435 with *P* = 0.000132, and OS HR = 0.445 with *P* = 0.000038, respectively, for ALI >18 versus ALI ≤18).

Next, we examined the relationship between other established or potential prognostic factors and the OS of immunotherapy-treated NSCLC patients (Supplementary Table S1, available at https://doi.org/10.1016/j.esmoop.2021.100254). Similar to the ALI, PD-L1 TPS and the NLR showed significant associations with OS only in case of PD-L1 inhibitor monotherapy (cohort A), but not in case of chemo-immunotherapy (cohort B). Additional significant factors were the ECOG PS for both cohorts and the line of immunotherapy treatment of cohort A, but not the patients' age or sex. Notably, based on the HR values, the ALI appeared to have a stronger effect than any of the other analyzed parameters in univariate analysis (Supplementary Table S1, available at https://doi.org/10.1016/j.esmoop.2021.100254).

To corroborate these results, we built a multivariable model including age (in years), sex, PD-L1 inhibitor monotherapy in the first line (yes/no, the latter referring to treatment in subsequent lines), ECOG PS (>1/1/0), and PD-L1 TPS (0/1-49/50+) and carried out a 10-fold cross-validation (Table 2). This analysis demonstrated that the prognostic effect of ALI on OS was considerable, increasing the Harrell's C-index for a model without ALI from 0.6444 to 0.6893 (*P* = 0.001 for difference between C-indices). In comparison, a model including NLR instead of ALI achieved a c-index of merely 0.6716. When adding both ALI and NLR to the prognostic model, the C-index was even smaller (C = 0.6867, Table 2) than for the proposed model with ALI without NLR, thus indicating that ALI has a higher prognostic value than NLR.

**Table 2:** Cross-validation of ALI as a prognostic and predictive marker in a multivariable model of overall survival after PD-L1 inhibitor monotherapy

	Cohort A (IO-monotherapy, *n* = 460)	
	HR (95% CI)	*P* value
ALI > 18	0.38 (0.23-0.60)	<0.0001
NLR > 5	0.92 (0.57-1.48)	0.73
Sex (male)	1.17 (0.86-1.57)	0.32
Age (years)	0.99 (0.98-1.01)	0.40
Line (first versus later)	1.26 (0.88-1.82)	0.21
ECOG PS = 1	2.05 (1.45-2.90)	<0.0001
ECOG PS >1	3.68 (2.38-5.67)	<0.0001
PD-L1 TPS 1-49	0.86 (0.60-1.24)	0.42
PD-L1 TPS ≥50	0.55 (0.36-0.84)	0.0062

Model variant	Harrel's C-index (95% CI)
no ALI/no NLR	0.6444 (0.6074-0.6813)
no ALI/(+) NLR	0.6716 (0.6356-0.7076)
(+) ALI/no NLR	0.6893 (0.6534-0.7252)
(+) ALI/(+) NLR	0.6867 (0.6508-0.7226)

Selected variables from Table 2 were used to build a multivariable Cox regression model of overall survival from start of immunotherapy followed by for 10-fold cross-validation. In the lower part, the Harrel's C-indices for the model with and without ALI and NLR are shown. The value of the C-index was highest for the model including only the ALI (0.6893, with a statistically significant difference from the value for a model without NLR and ALI 0.6444, with *P* = 0.0010)

ALI, advanced lung cancer inflammation index, CI, confidence interval; ECOG PS, Eastern Cooperative Oncology Group performance status; HR, hazard ratio; IO, immunotherapy; NLR, neutrophil-to-lymphocyte ratio, PD-L1, programmed death-ligand 1; TPS, tumor proportion score.

### Effect of ALI on ORR, DCR and TOT

ALI was significantly associated with ORR, DCR, and TOT in NSCLC patients treated with PD-L1 monotherapy (cohort A): for ALI >18 versus ALI ≤18, ORR was 36% versus 24% (*P* = 0.0080), DCR 65% versus 46% (*P* < 0.001), and the TOT HR = 0.52 (CI 0.41-0.65, *P* < 0.001, Table 3). Significant associations were observed also for NLR and PD-L1 TPS (Table 3). In contrast, none of these parameters showed significant associations with ORR, DCR, and TOT for patients treated with chemo-immunotherapy (Table 3), similar to what had already been observed for OS (Table 2).

**Table 3:** Relationship of ALI with response rate, disease control rate, time-on-treatment and of immunotherapy-treated NSCLC patients

	Cohort A (IO-monotherapy, *n* = 460)		Cohort B (Chemo-immunotherapy, *n* = 212)	
	ORR %	*P*	ORR %	*P*
ALI > 18	36 versus 24	0.008	43 versus 42	0.90
NLR < 5	37 versus 23	0.001	43 versus 42	0.84
PD-L1 TPS^a^	44 versus 22 versus 14	<0.001	50 versus 41 versus 38	0.43

	DCR %	*P*	DCR %	*P*
ALI > 18	65 versus 46	<0.001	77 versus 74	0.60
NLR < 5	66 versus 45	<0.001	75 versus 75	0.91
PD-L1 TPS^a^	68 versus 41 versus 49	<0.001	77 versus 73 versus 78%	0.74

	TOT HR (95% CI)	*P*	TOT HR (95% CI)	*P*
ALI > 18	0.52 (0.41-0.65)	<0.001	0.64 (0.42-0.97)	0.034
NLR < 5	0.56 (0.44-0.70)	<0.001	0.82 (0.54-1.23)	0.33
PD-L1 TPS 1-49%	1.08 (0.78-1.48)	0.65	0.92 (0.56-1.53)	0.76
PD-L1 TPS ≥50%	0.66 (0.48-0.91)	0.011	0.88 (0.49-1.57)	0.66

The association of each factor with overall survival (OS) was analyzed with a univariable Cox regression. ALI and the NLR were dichotomized at the bibliographic cut-offs of 18 and 5 respectively, which corresponded to the median values of our untreated patients (see Patients and methods in the main text). The PD-L1 tumor proportion score was divided in three categories (0/1-49/50+) and included as a categorical variable with 0 as the reference. The ECOG PS was divided in 0/1/>1 and included as a categorical variable with 0 as the reference.

ALI, advanced lung cancer inflammation index, CI, confidence interval; DCR, disease control rate; ECOG PS, Eastern Cooperative Oncology Group performance status; HR, hazard ratio; IO, immunotherapy; NLR, neutrophil-to-lymphocyte ratio, ORR, objective response rate; PD-L1, programmed death-ligand 1; TPS, tumor proportion score.

a PD-L1 TPS ≥50% versus 1-49% versus <1%.

### ALI versus other laboratory and algorithmic biomarkers: dNLR, LDH, LIPI, EPSILoN

In order to assess the potential utility of ALI compared with other readily available laboratory markers and scores of immunotherapy in NSCLC, such as dLNR, LDH, LIPI, EPSILoN, we compared their performance in the subset of patients with available data (206/460 of cases in cohort A, and 107/212 in cohort B, for which all laboratory results as well as data on PFS were available, Supplementary Table S2, available at https://doi.org/10.1016/j.esmoop.2021.100254). ALI performed similarly well as the more complex score EPSILoN (which incorporates the NLR, serum LDH as well as three clinical parameters, namely ECOG PS, smoking history, and presence of liver metastases), and showed a numerically stronger association with PFS and OS than other laboratory parameters, namely the dNLR, LDH, and LIPI (Supplementary Table S3, available at https://doi.org/10.1016/j.esmoop.2021.100254). Again, strong associations were observed in case of treatment with PD-L1 monotherapy only, while for chemo-immunotherapy, associations were absent or of marginal significance, with the only exception being the association between LDH and OS (Supplementary Table S3, available at https://doi.org/10.1016/j.esmoop.2021.100254).

### Potential clinical utility of ALI for the decision between PD-L1 monotherapy and chemo-immunotherapy for NSCLC with PD-L1 TPS ≥50%

Finally, we examined whether the observed association of ALI with efficacy of PD-L1 monotherapy could be exploited clinically. In current clinical practice, PD-L1 inhibitor monotherapy is only offered to newly diagnosed patients with high PD-L1 TPS ≥50%, for which chemo-immunotherapy is also approved, but established criteria for the choice between the two options are lacking. To explore the potential utility of ALI in this setting, we examined the OS and TOT of all patients with PD-L1 TPS ≥50% and first-line PD-L1 monotherapy (*n* = 156, 150 pembrolizumab, 5 nivolumab, 1 atezolizumab) or chemo-immunotherapy (*n* = 38), according to the ALI score. Again, ALI (dichotomized at 18) could separate the OS curves of patients treated with PD-L1 monotherapy (HR = 0.36, median OS 35 versus 18 months, *P* < 0.0001), but not the curves of patients treated with chemo-immunotherapy (*P* = 0.24, median OS not reached, Figure 2D and Supplementary Figure S1, available at https://doi.org/10.1016/j.esmoop.2021.100254). Similarly, the ALI score could separate the TOT curves of patients treated with PD-L1 monotherapy (HR = 0.53, median TOT 16 versus 4 months, *P* = 0.004), but not those of patients treated with chemo-immunotherapy (*P* = 0.94, median TOT 20.2 months, Figure 2E and Supplementary Figure S1, available at https://doi.org/10.1016/j.esmoop.2021.100254). Thus, regarding both endpoints, for NSCLC patients with PD-L1 TPS ≥50% treated with first-line PD-L1 inhibitor monotherapy, a high ALI score (>18) was able to distinguish a favorable subset with outcomes similar to those who received chemo-immunotherapy (Figure 2D and E).

## Discussion

A large number of less or more complex clinical and laboratory biomarkers have been evaluated in an effort to optimize the use of immunotherapy in patients with advanced NSCLC. Immunohistochemical expression of PD-L1, albeit suboptimal, has been the most widely adopted in clinical practice to date because of its relatively simple evaluation method and moderate to high reproducibility.^1^^,^^13^ Nevertheless, the choice between ICI monotherapy and chemo-immunotherapy, as first-line treatment, still remains controversial, especially in cases of high PD-L1 expression (TPS ≥50%), where both treatment modalities are currently approved by the Food and Drug Administration (FDA) and European Medicines Agency (EMA)^2^, ^3^, ^4^ and the choice between them remains mainly clinical. In the current analysis, we show that a simple combination of clinical markers assessed in routine clinical practice (ALI score, comprising body mass index, serum albumin levels, and NLR) was able to predict clinical benefit from ICI monotherapy both in the whole cohort treated with ICIs in any line of treatment (cohort A, Figure 2A) and in the subgroup of patients receiving ICI monotherapy (mostly pembrolizumab) as first line (Figure 2D and E). This observation carries clinical value, since ALI >18 is able to detect a subgroup of patients with high PD-L1 expression for whom ICI monotherapy has similar efficacy to chemo-immunotherapy and thus could be spared from the addition of unnecessary and potentially hazardous chemotherapy. In this context, ALI could serve as a useful stratification factor for trials comparing chemotherapy + ICI with ICI monotherapy, such as the ongoing PERSEE trial, comparing pembrolizumab plus chemotherapy with pembrolizumab alone in patients with PD-L1 TPS ≥50% (NCT04547504).

In our analysis, ALI was not only prognostic, but also predictive of the benefit from ICI monotherapy, since its predictive effect for chemotherapy efficacy was less pronounced (Figure 2C) and the test for interaction according to the type of treatment was highly statistically significant in favor of immunotherapy. Moreover, ALI outperformed a number of other less or more complex potential biomarkers of activity, including the NLR, PD-L1 TPS, LIPI, and EPSILoN index. The fact that ALI consists of variables that are easily collected at the daily clinical routine and that it possesses higher predictive capacity than the other tested markers renders it an appealing algorithmic tool for treatment guidance in clinical practice. NLR has also been associated with clinical outcomes in advanced NSCLC^27^^,^^28^ and has prognostic value in NSCLC patients treated with nivolumab^22^ and pembrolizumab,^29^ but this marker alone rather reflects systemic inflammation of the host and can be affected by extrinsic factors, such as infections or granulocyte colony-stimulating factor administration. A recent study evaluated its combination with either PD-L1 or LDH in patients with high PD-L1 expression but showed the prognostic and not the predictive capacity of the combinations, as there was no control arm receiving chemotherapy.^30^ Mezquita et al.^23^ recently reported that the LIPI, incorporating dNLR and LDH, was correlated with worse outcomes for ICI, but not for chemotherapy, suggesting that LIPI can serve as a predictive tool when selecting ICI treatment,^23^ although a subsequent pooled analysis did not confirm this specificity for immunotherapy efficacy.^31^ In our analysis, LIPI score had a weaker effect on predicting ICI efficacy compared with ALI (HR = 0.57 and 0.45, respectively, in multivariate analysis, Supplementary Table S3, available at https://doi.org/10.1016/j.esmoop.2021.100254). Finally, the more complex EPSILoN index, which incorporates the NLR, serum LDH, as well as three clinical parameters, namely ECOG PS, smoking history, and presence of liver metastases, had equally high performance to ALI (HR = 0.43 and 0.45, respectively, in multivariate analysis, Supplementary Table S3, available at https://doi.org/10.1016/j.esmoop.2021.100254), but ALI is much simpler and easier to calculate.

The current work suggests that ALI loses its predictive capacity when chemotherapy is added to immunotherapy. This observation is similar to that of other markers of ICI efficacy: it is well established, for example, that PD-L1 TPS is not predictive of efficacy from chemo-immunotherapy combinations, as evidenced across multiple randomized trials.^3^^,^^4^^,^^7^ Similarly, *post hoc* analyses of data from prospective randomized trials show that tumor mutational burden correlates well with benefit from PD-L1 inhibitor monotherapy, but loses its predictive ability in case of chemo-immunotherapy.^32^^,^^33^ It has been suggested that the addition of cytotoxic agents abolishes the capacity of PD-L1 to reflect the level of engagement of PD-L1 inhibitors with their corresponding targets on the surface of cancer or immune cells.^34^ Irrespective of the potential mechanism, identification of robust biomarkers of activity of chemo-immunotherapy remains an unmet medical need.

To our knowledge, this is the first large comparative study evaluating the prognostic and predictive capacity of ALI in patients with advanced NSCLC, treated with immunotherapy, chemotherapy, or both, in relation to other potential biomarkers of ICI efficacy. Still, the current work harbors some limitations: being retrospective in nature, a number of clinical or laboratory variables were not available for all patients (Figure 1), as well as subsequent treatments, thus limiting the number of cases available for cross-biomarker analysis. Second, data were collected from a large number of cancer centers in Greece, which might have allowed a substantial level of heterogeneity and might have enabled a selection bias, according to the treating physician's choice. This inherent bias to select patients who are more likely to be in a good physical condition may also account for the slightly younger median age of patients at diagnosis in our cohorts (63-67 years). Finally, the number of patients with PD-L1 >50%, receiving first-line ICI monotherapy and chemo-immunotherapy was relatively small (*n* = 160 and 55, respectively), compared with the total population; still, the results of the analysis for these subgroups clearly confirm that ALI is able to identify patients who derive equal benefit from immunotherapy alone.

In conclusion, our comparative analysis shows that ALI is a robust prognostic and predictive biomarker of immunotherapy efficacy, when ICIs are administered as monotherapy, but not in combination with chemotherapy. ALI outperformed other widely used parameters, such as the PD-L1 TPS, NLR, and LIPI. Importantly, for PD-L1-high patients, an ALI score >18 may assist in the selection of patients who do not need addition of chemotherapy.

## References

[1] Remon J., Passiglia F., Ahn M.J. (2020). Immune checkpoint inhibitors in thoracic malignancies: review of the existing evidence by an IASLC expert panel and recommendations. J Thorac Oncol.

[2] Reck M., Rodríguez-Abreu D., Robinson A.G. (2016). Pembrolizumab versus chemotherapy for PD-L1-positive non-small-cell lung cancer. N Engl J Med.

[3] Gandhi L., Rodríguez-Abreu D., Gadgeel S. (2018). Pembrolizumab plus chemotherapy in metastatic non-small-cell lung cancer. N Engl J Med.

[4] Paz-Ares L., Luft A., Vicente D. (2018). Pembrolizumab plus chemotherapy for squamous non-small-cell lung cancer. N Engl J Med.

[5] Herbst R.S., Giaccone G., de Marinis F. (2020). Atezolizumab for first-line treatment of PD-L1-selected patients with NSCLC. N Engl J Med.

[6] West H., McCleod M., Hussein M. (2019). Atezolizumab in combination with carboplatin plus nab-paclitaxel chemotherapy compared with chemotherapy alone as first-line treatment for metastatic non-squamous non-small-cell lung cancer (IMpower130): a multicentre, randomised, open-label, phase 3 trial. Lancet Oncol.

[7] Socinski M.A., Jotte R.M., Cappuzzo F. (2018). Atezolizumab for first-line treatment of metastatic nonsquamous NSCLC. N Engl J Med.

[8] Brahmer J., Reckamp K.L., Baas P. (2015). Nivolumab versus docetaxel in advanced squamous-cell non-small-cell lung cancer. N Engl J Med.

[9] Herbst R.S., Baas P., Kim D.W. (2016). Pembrolizumab versus docetaxel for previously treated, PD-L1-positive, advanced non-small-cell lung cancer (KEYNOTE-010): a randomised controlled trial. Lancet.

[10] Borghaei H., Paz-Ares L., Horn L. (2015). Nivolumab versus docetaxel in advanced nonsquamous non-small-cell lung cancer. N Engl J Med.

[11] Rittmeyer A., Barlesi F., Waterkamp D. (2017). Atezolizumab versus docetaxel in patients with previously treated non-small-cell lung cancer (OAK): a phase 3, open-label, multicentre randomised controlled trial. Lancet.

[12] Chan T.A., Yarchoan M., Jaffee E. (2019). Development of tumor mutation burden as an immunotherapy biomarker: utility for the oncology clinic. Ann Oncol.

[13] Bodor J.N., Boumber Y., Borghaei H. (2020). Biomarkers for immune checkpoint inhibition in non-small cell lung cancer (NSCLC). Cancer.

[14] Thompson J.C., Hwang W.T., Davis C. (2020). Gene signatures of tumor inflammation and epithelial-to-mesenchymal transition (EMT) predict responses to immune checkpoint blockade in lung cancer with high accuracy. Lung Cancer.

[15] Gelsomino F., Lamberti G., Parisi C. (2019). The evolving landscape of immunotherapy in small-cell lung cancer: a focus on predictive biomarkers. Cancer Treat Rev.

[16] Hanahan D., Weinberg R.A. (2011). Hallmarks of cancer: the next generation. Cell.

[17] Baracos V.E., Martin L., Korc M., Guttridge D.C., Fearon K.C.H. (2018). Cancer-associated cachexia. Nat Rev Dis Primers.

[18] Prelaj A., Ferrara R., Rebuzzi S.E. (2019). EPSILoN: a prognostic score for immunotherapy in advanced non-small-cell lung cancer: a validation cohort. Cancers (Basel).

[19] Jafri S.H., Shi R., Mills G. (2013). Advance lung cancer inflammation index (ALI) at diagnosis is a prognostic marker in patients with metastatic non-small cell lung cancer (NSCLC): a retrospective review. BMC Cancer.

[20] Hua X., Chen J., Wu Y., Sha J., Han S., Zhu X. (2019). Prognostic role of the advanced lung cancer inflammation index in cancer patients: a meta-analysis. World J Surg Oncol.

[21] Travis W.D., Brambilla E., Nicholson A.G. (2015). The 2015 World Health Organization Classification of Lung Tumors: impact of genetic, clinical and radiologic advances since the 2004 classification. J Thorac Oncol.

[22] Diem S., Schmid S., Krapf M. (2017). Neutrophil-to-lymphocyte ratio (NLR) and platelet-to-lymphocyte ratio (PLR) as prognostic markers in patients with non-small cell lung cancer (NSCLC) treated with nivolumab. Lung Cancer.

[23] Mezquita L., Auclin E., Ferrara R. (2018). Association of the lung immune prognostic index with immune checkpoint inhibitor outcomes in patients with advanced non-small cell lung cancer. JAMA Oncol.

[24] Huang Bartlett C., Mardekian J., Cotter M.J. (2020). Concordance of real-world versus conventional progression-free survival from a phase 3 trial of endocrine therapy as first-line treatment for metastatic breast cancer. PLoS One.

[25] Ma X., Nussbaum N.C., Magee K. (2019). Comparison of real-world response rate (rwRR) to RECIST-based response rate in patients with advanced non-small cell lung cancer (aNSCLC). Ann Oncol.

[26] Zhou X.H., Li C.M., Yang Z. (2008). Improving interval estimation of binomial proportions. Philos Trans A Math Phys Eng Sci.

[27] Mandaliya H., Jones M., Oldmeadow C., Nordman I.I. (2019). Prognostic biomarkers in stage IV non-small cell lung cancer (NSCLC): neutrophil to lymphocyte ratio (NLR), lymphocyte to monocyte ratio (LMR), platelet to lymphocyte ratio (PLR) and advanced lung cancer inflammation index (ALI). Transl Lung Cancer Res.

[28] Alessi J, Ricciuti B, Alden S, et al. Peripheral blood derived neutrophil-to-lymphocyte ratio (dNLR) impacts tumor-infiltrating immune cells and clinical outcomes to first-line pembrolizumab in NSCLC with high PD-L1 (≥50%) expression. Presented at WCLC 2020, Poster P14.21.

[29] Hasegawa T., Yanagitani N., Utsumi H. (2019). Association of high neutrophil-to-lymphocyte ratio with poor outcomes of pembrolizumab therapy in high-PD-L1-expressing non-small cell lung cancer. Anticancer Res.

[30] Banna G.L., Signorelli D., Metro G. (2020). Neutrophil-to-lymphocyte ratio in combination with PD-L1 or lactate dehydrogenase as biomarkers for high PD-L1 non-small cell lung cancer treated with first-line pembrolizumab. Transl Lung Cancer Res.

[31] Kazandjian D., Gong Y., Keegan P., Pazdur R., Blumenthal G.M. (2019). Prognostic value of the lung immune prognostic index for patients treated for metastatic non-small cell lung cancer. JAMA Oncol.

[32] Herbst R.S., Lopes G., Kowalski D.M. (2019). Association between tissue TMB (tTMB) and clinical outcomes with pembrolizumab monotherapy (pembro) in PD-L1-positive advanced NSCLC in the KEYNOTE-010 and -042 trials. Ann Oncol.

[33] Paz-Ares L., Langer C.J., Novello S. (2019). Pembrolizumab (pembro) plus platinum-based chemotherapy (chemo) for metastatic NSCLC: tissue TMB (tTMB) and outcomes in KEYNOTE-021, 189, and 407. Ann Oncol.

[34] Salmaninejad A., Valilou S.F., Shabgah A.G. (2019). PD-1/PD-L1 pathway: basic biology and role in cancer immunotherapy. J Cell Physiol.

