# Supplementary material for: Association of the advanced lung cancer inflammation index (ALI) with immune checkpoint inhibitor efficacy in patients with advanced non-small-cell lung cancer
Source: ESMO Open. 2021 Sep 1;6(5):100254. doi: 10.1016/j.esmoop.2021.100254 (PMC8417333; doi:10.1016/j.esmoop.2021.100254)
Supplement: Supplementary Figure S1 [file mmc1.pdf]

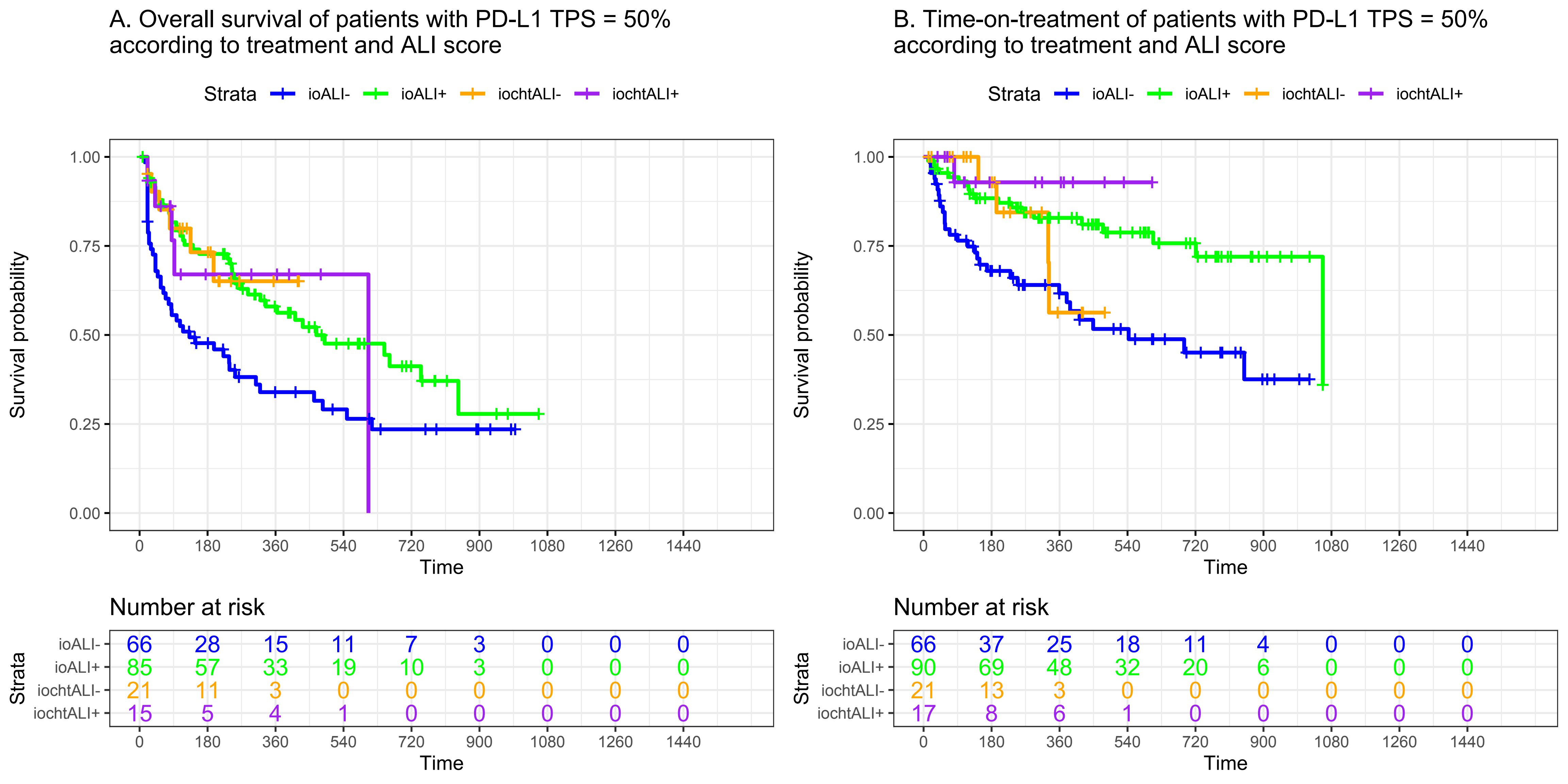
A. Overall survival of patients with PD-L1 TPS = 50%
according to treatment and ALI score
Strata ioALI- ioALI+ iochtALI- iochtALI+
Survival probability
1.00
0.75
0.50
0.25
0.00
0 180 360 540 720 900 1080 1260 1440
Time
Number at risk
ioALI- 66 28 15 11 7 3 0 0 0
ioALI+ 85 57 33 19 10 3 0 0 0
iochtALI- 21 11 3 0 0 0 0 0 0
iochtALI+ 15 5 4 1 0 0 0 0 0
Strata
0 180 360 540 720 900 1080 1260 1440
Time
B. Time-on-treatment of patients with PD-L1 TPS = 50%
according to treatment and ALI score
Strata ioALI- ioALI+ iochtALI- iochtALI+
Survival probability
1.00
0.75
0.50
0.25
0.00
0 180 360 540 720 900 1080 1260 1440
Time
Number at risk
ioALI- 66 37 25 18 11 4 0 0 0
ioALI+ 90 69 48 32 20 6 0 0 0
iochtALI- 21 13 3 0 0 0 0 0 0
iochtALI+ 17 8 6 1 0 0 0 0 0
Strata
0 180 360 540 720 900 1080 1260 1440
Time
