# Supplementary material for: Association of the advanced lung cancer inflammation index (ALI) with immune checkpoint inhibitor efficacy in patients with advanced non-small-cell lung cancer
Source: ESMO Open. 2021 Sep 1;6(5):100254. doi: 10.1016/j.esmoop.2021.100254 (PMC8417333; doi:10.1016/j.esmoop.2021.100254)
Supplement: Supplementary Table S1 [file mmc2.docx]

**Supplementary Table 1. Relationship of established and potential prognostic factors with overall survival of immunotherapy-treated NSCLC patients**

The association of each factor with OS under immunotherapy alone (IO-monotherapy) or chemoimmunotherapy was analyzed with a univariable Cox regression. ALI and the NLR were dichotomized at the bibliographic cut-offs of 18 and 5 respectively, which corresponded to the median values of our untreated patients (s. Methods). The PD-L1 tumor proportion score (TPS) was divided in 3 categories (<1%/1-49%/≥50%) and included as a categorical variable with <1% as the reference. The ECOG performance status (PS) was divided in 0/1/>1 and included as a categorical variable with 0 as the reference. The body mass index (BMI), weight, height and serum albumin were dichotomized at the median values of patients in our experimental cohort AB (Table 1).

|  | cohort A  (IO-monotherapy, n=460) | | cohort B  (chemoimmunotherapy, n=212) | |
| --- | --- | --- | --- | --- |
|  | HR (95% CI) | P-value | HR (95% CI) | P-value |
| age | 1.00 (0.99-1.02) | 0.82 | 1.03 (0.99-1.04) | 0.28 |
| sex | 0.99 (0.74-1.33_ | 0.97 | 1.68 (0.93-3.02) | 0.08 |
| ALI > 18 | 0.41 (0.31-0.54) | <0.0001 | 0.68 (0.40-1.16) | 0.16 |
| NLR < 5 | 0.48 (0.37-0.63) | <0.0001 | 0.88 (0.52-1.49) | 0.63 |
| PD-L1 TPS 1-49 | 0.91 (0.64-1.30) | 0.61 | 1.14 (0.59-2.20) | 0.70 |
| PD-L1 TPS ≥ 50 | 0.52 (0.36-0.75) | <0.0001 | 0.93 (0.43-2.02) | 0.86 |
| ECOG PS =1 | 2.30 (1.64-3.22) | <0.0001 | 2.24 (1.10-4.56) | 0.026 |
| ECOG PS >1 | 4.12 (2.72-6.24) | <0.0001 | 9.50 (4.45-20.25) | <0.0001 |
| Line (first *vs.* later) | 1.43 (1.23-1.68) | <0.0001 | --- |  |
| BMI >25 | 0.69 (0.52-0.91) | 0.008 | 0.65 (0.67-1.92) | 0.65 |
| Weight >72 | 0.69 (0.52-0.90) | 0.007 | 1.35 (0.79-2.31) | 0.27 |
| Height >1.70 | 1.12 (0.86-1.47) | 0.41 | 1.65 (0.96-2.83) | 0.07 |
| Albumin >3.9 | 0.46 (0.34-0.62) | <0.0001 | 0.40 (0.23-0.69) | 0.001 ^1^ |

^1.^ Spearman r=-0.35, p=1.98*10^-7^ for the correlation between serum albumin and ECOG PS in cohort B.
