# Supplementary material for: Association of the advanced lung cancer inflammation index (ALI) with immune checkpoint inhibitor efficacy in patients with advanced non-small-cell lung cancer
Source: ESMO Open. 2021 Sep 1;6(5):100254. doi: 10.1016/j.esmoop.2021.100254 (PMC8417333; doi:10.1016/j.esmoop.2021.100254)
Supplement: Supplementary Table S2 [file mmc3.docx]

**Supplementary Table 2. The subset of patients (A1, B1) with available data for additional biomarkers**

|  |  | PD-(L)1 inhibitor monotherapy  (A1, n=206) | Chemoimmuno-therapy  (B1, n=107) |
| --- | --- | --- | --- |
| Age at diagnosis, median (SD) |  | 65 (11) | 63 (9) |
| Sex, % male (n) |  | 61% (125) | 56% (60) |
| ECOG PS, % (n) | PS = 0 | 23% (48) | 41% (44) |
|  | PS = 1 | 68% (139) | 59% (63) |
|  | PS = 2 | 9% (19) | - |
| Histology, % (n) | adenocarcinoma | 73% (151) | 94% (101) |
|  | squamous carcinoma | 18% (36) | 6% (6) |
|  | other | 9% (19) | - |
| PD-L1 expression, % (n) | negative (TPS<1%) | 11% (23) | 31% (33) |
|  | TPS 1-49% | 37% (76) | 51% (54) |
|  | TPS ≥50% | 52% (107) | 19% (20) |
| Line of treatment, % (n) | first line | 32% (65) | 100% (107) ^1^ |
|  | second-and-beyond | 68% (141) |  |
| Baseline characteristics: |  |  |  |
| NLR, median (SD) |  | 5.7 (9.3) | 7.3 (7.2) |
| Height m, median (SD) |  | 1.70 (0.09) | 1.70 (0.09) |
| Weight kg, median (SD) |  | 70 (16.8) | 70 (16.6) |
| BMI, median (SD) |  | 24 (5.0) | 24 (4.3) |
| Albumin, median (SD) |  | 3.6 (0.6) | 4.1 (0.5) |
| ALI, median (SD) |  | 14.5 (16.5) | 12.4 (11.8) |
| dNLR, median (SD) |  | 2.96 (2.3) | 4.44 (7.8) |
| LDH, median (SD) |  | 255 (547) | 235 (294) |
| CRP, median (SD) |  | 30 (62) | 19 (109) |
| pack-years, median (SD) |  | 40 (24) | 36 (28) |
| liver metastases present |  | 23% (47) | 18% (19) |
| PIOS, median (SD) |  | 0.17 (1.8) | 0.25 (0.19) |
|  |  |  |  |
| Clinical outcome: |  |  |  |
| ORR, % (95% CI) |  | 24% (19-30) | 45% (36-54) |
| DCR, % (95%CI) |  | 54% (48-61) | 79% (71-86) |
| PFS, median (95% CI), months ^2^ |  | 3.3 (2.1-4.4) | 8.0 (6.4-.9.7) |
| OS, median (95% CI), months |  | 11.5 (8.4-14.7) | 25.6 (8.8-42.4) |

ECOG PS: Eastern Cooperative Oncology Group performance status; ANC: absolute neutrophil count; ALC: absolute lymphocyte count; (d)NLR: (derived) blood neutrophil-to-lymphocyte ratio; BMI: body-mass index; ALI: advanced lung cancer inflammation index; ORR: objective response rate; DCR: disease control rate; PFS: progression-free survival; OS: overall survival; 95% CI: 95% confidence interval.

^1.^ previous stage 3 disease in 13% (n=14) in cohort B1.

^2.^ PFS data available for 206/460 (45%) patients in cohort A, 107/212 (50%) patients in cohort B, and 313/672 (47%) patients in cohort AB.
