# Supplementary material for: Association of the advanced lung cancer inflammation index (ALI) with immune checkpoint inhibitor efficacy in patients with advanced non-small-cell lung cancer
Source: ESMO Open. 2021 Sep 1;6(5):100254. doi: 10.1016/j.esmoop.2021.100254 (PMC8417333; doi:10.1016/j.esmoop.2021.100254)
Supplement: Supplementary Table S3 [file mmc4.docx]

**Supplementary Table 3. Relative strength of ALI and other laboratory markers and/or scores of immunotherapy efficacy in NSCLC**

The association of each factor with OS was analyzed with a univariable Cox regression. ALI and the NLR were dichotomized at the bibliographic cut-offs of 18 and 5 respectively, which corresponded to the median values of our untreated patients (s. Methods). LIPI (3 risk categories) and EPSILON (3 risk categories) were included as ordinal variables. The dNLR was dichotomized at the bibliographic cut-off of 3. The LDH was dichotomized at median value in our cohort (suppl. Table 1).

|  | cohort A  (IO-monotherapy, n=206) | | cohort B  (chemoimmunotherapy, n=107) | |
| --- | --- | --- | --- | --- |
|  | OS HR (95% CI) | p-value | OS HR (95% CI) | p-value |
| ALI > 18 | 0.45 (0.30-0.65) | <0.0001 | 1.51 (0.63-3.65) | 0.36 |
| dNLR < 3 | 0.59 (0.42-0.84) | 0.004 | 2.51 (1.06-5.92) | 0.036 |
| LDH < 248 (median) | 0.53 (0.37-0.76) | 0.0005 | 0.40 (0.18-0.93) | 0.034 |
| LIPI low | 0.57 (0.45-0.73) | <0.0001 | 0.79 (0.34-1.83) | 0.58 |
| EPSILoN low | 0.43 (0.30-0.61) | <0.0001 | 0.59 (0.22-1.59) | 0.30 |
|  |  |  |  |  |
|  | PFS HR (95% CI) | p-value | PFS HR (95% CI) | p-value |
| ALI > 18 | 0.54 (0.39-0.74) | 0.0002 | 1.04 (0.61-1.77) | 0.89 |
| dNLR < 3 | 0.63 (0.46-0.85) | 0.003 | 1.49 (0.90-2.45) | 0.12 |
| LDH < 248 (median) | 0.62 (0.46-0.84) | 0.002 | 0.66 (0.40-1.08) | 0.10 |
| LIPI low | 0.64 (0.52-0.78) | <0.0001 | 0.79 (0.50-1.26) | 0.33 |
| EPSILoN low | 0.58 (0.42-0.81) | 0.001 | 1.03 (0.59-1.79) | 0.93 |
|  |  |  |  |  |
